# Lung Deposition Analyses of Inhaled Toxic Aerosols in Conventional and Less Harmful Cigarette Smoke: A Review

**DOI:** 10.3390/ijerph10094454

**Published:** 2013-09-23

**Authors:** Clement Kleinstreuer, Yu Feng

**Affiliations:** 1 Department of Mechanical and Aerospace Engineering, North Carolina State University, Raleigh, NC 27695, USA; E-Mail: yfeng4@ncsu.edu; 2 Joint UNC-NCSU Department of Biomedical Engineering, North Carolina State University, Raleigh, NC 27695, USA

**Keywords:** conventional or less harmful cigarettes, aerosol toxicology, cigarette smoke droplet/vapor deposition, second-hand smoke, impact analysis, computational fluid-particle dynamics

## Abstract

Inhaled toxic aerosols of conventional cigarette smoke may impact not only the health of smokers, but also those exposed to second-stream smoke, especially children. Thus, less harmful cigarettes (LHCs), also called potential reduced exposure products (PREPs), or modified risk tobacco products (MRTP) have been designed by tobacco manufacturers to focus on the reduction of the concentration of carcinogenic components and toxicants in tobacco. However, some studies have pointed out that the new cigarette products may be actually more harmful than the conventional ones due to variations in puffing or post-puffing behavior, different physical and chemical characteristics of inhaled toxic aerosols, and longer exposure conditions. In order to understand the toxicological impact of tobacco smoke, it is essential for scientists, engineers and manufacturers to develop experiments, clinical investigations, and predictive numerical models for tracking the intake and deposition of toxicants of both LHCs and conventional cigarettes. Furthermore, to link inhaled toxicants to lung and other diseases, it is necessary to determine the physical mechanisms and parameters that have significant impacts on droplet/vapor transport and deposition. Complex mechanisms include droplet coagulation, hygroscopic growth, condensation and evaporation, vapor formation and changes in composition. Of interest are also different puffing behavior, smoke inlet conditions, subject geometries, and mass transfer of deposited material into systemic regions. This review article is intended to serve as an overview of contributions mainly published between 2009 and 2013, focusing on the potential health risks of toxicants in cigarette smoke, progress made in different approaches of impact analyses for inhaled toxic aerosols, as well as challenges and future directions.

## 1. Introduction

Cigarette smoke is a complex assemblage of liquid droplets, *i.e.*, particulate phase, suspended in a mixture of gases and vapors. The toxicants are dissolved in droplets or vapors or both [1], where the droplets range in variable mean diameter from the nano- (d_p_ < 100 nm) into the sub-micrometer scale (d_p_ = 0.1−1 μm) [2]. Cigarette smoking causes a variety of diseases, primarily lung tumors and deficiencies of the respiratory and cardiovascular system [3]. So far, more than 6,000 compounds have been found in cigarette smoke [4], at least 69 of which are known or probable human carcinogens [5]. More recently, the FDA [6] listed tobacco-smoke constituents which are directly harmful (*i.e.*, all carcinogens and toxicants) and potentially harmful (e.g., nicotine being not only a reproductive toxicant but also addictive). 

Smoking causes damage primarily to the respiratory tract where almost half of all smoking-related health effects materialize. Specifically, lung cancer and chronic obstructive pulmonary disease (COPD) are two potential fatal outcomes [7]. Concerning second-hand, or side-stream, smoke (*i.e.*, environmental tobacco smoke), the high concentration of CO and tar may be harmful as well [8,9]. To reduce the potential health risks of cigarette smoking, manufacturers world-wide have introduced and marketed a variety of “less harmful cigarette (LHC)” products [10] in the past decades. However, many papers found that the new cigarettes may be more harmful than the conventional ones [11,12,13], due to compensatory puffing behavior and other differences. 

To evaluate the potential health risks of new emerging cigarette products on humans, results from separate or combined experimental, clinical and numerical studies can be considered. Although epidemiologic and naturalistic analyses based on experiments and clinical tests provide considerable support for the relationship between cigarette-smoke exposure and pulmonary and cardiovascular diseases, the exposure-dose and dose relationship between cigarette smoke and these diseases are still poorly understood. Hence, in addition to experimental and clinical investigations, a realistic and experimentally validated computer simulation model for impact analyses is desirable. It should be able to predict inhaled tobacco smoke droplet/vapor and toxicant deposition for a set of realistic inlet conditions on a subject-specific basis. Furthermore, in conjunction with a multi-compartment model [14,15] for deposited constituent mass transfer into organs, it is a valuable and cost-effective tool for toxicologists and others to establish dose-response relationships and generate new physical insight and reliable, quantitative data sets. 

In this review, we begin with a brief discussion of the toxicology of cigarette smoke, followed by the introduction of different manufacturing approaches and tobacco products, as well as their potential health risks to humans, especially to the most vulnerable population groups, *i.e.*, children and seniors. Next, existing quantitative and qualitative approaches are presented for different parameter impacts on the potential health risks of cigarette smoking. The last section is devoted to challenges and future research directions.

## 2. Cigarette Toxicity and Vulnerable Population Groups

### 2.1. Toxicants and Carcinogens in Conventional Cigarette Smoke

Toxic smoke-particulate matter and vapor inhalation can cause adverse acute and chronic effects on both respiratory and cardiovascular systems, affecting especially the most vulnerable population groups, *i.e.*, children and the elderly as well as patients with asthma [16] and/or COPD [17,18]. Other than pathological effects on human respiratory systems, cigarette smoke also increases the risk of many diseases [19,20,21,22], including breast cancer [23,24,25]. Cigarette smoke is a complex, reactive mixture. As mentioned, more than 6,000 compounds were reported to appear in cigarette smoke [4]. By using an FTC (Federal Trade Commission) smoking machine (35 mL/puff, puff duration 2.0 s), Rodgman and Perfetti [4] listed the key constituents of mainstream smoke. The majority of components can be found in the particulate phase (*i.e.*, droplets or particles), while the vapor phase contains approximately 400 to 500 compounds of which 300 can be classified as semi-volatiles [26,27]. 

Cigarette mainstream smoke is the smoke emerging from the mouth end of a cigarette during puffing [28]. Mainstream smoke consist of an aerosol containing liquid droplets (particulate phase) suspended in the gas-vapor phase, which is generated by overlapping burning, pyrolysis, pyrosynthesis, distillation, sublimation, and condensation processes [29]. Temperatures of 860–900 °C are attained in the burning zone for mainstream smoke. More than 60 known carcinogens have been detected in mainstream smoke and most of the same carcinogens are present in sidestream smoke [30,31], e.g., acetaldehyde, benzene, BaP, 1,3-butadiene, formaldehyde, ethylene oxide, cadmium, 4-aminobiphenyl NNN, and NNK. Discussions of the toxicities can be found in [27].

Second-hand smoke (SHS), also known as environmental tobacco smoke (ETS), is the sidestream smoke emitted from the burning of the tip of a cigarette [32,33]. There are more than 3,000 chemicals in SHS and more than 60 of them have been identified as toxicants or carcinogens [6,30,31,34]. Temperatures of 500–600 °C are attained in the burning zone for sidestream smoke. Second-hand tobacco smoke (SHS) is a major source of indoor air pollution in the US [35]. SHS causes a lot of harmful health effects, including respiratory illness, asthma, otitis media, sudden infant death syndrome, vascular dysfunction, and predisposition toward cardiovascular disease and cancer [17,36,37,38,39]. It is also reported that inhaled fresh sidestream cigarette smoke is approximately four times more toxic per gram total particulate matter (TPM) than mainstream cigarette smoke [40,41]. 

### 2.2. Less Harmful Cigarette Products

Government and public health experts have long advocated the development of less harmful cigarettes (LHCs) to reduce the health risk of smoking [42]. Less harmful cigarettes (LHCs), also called potential reduced exposure products (PREPs), tobacco harm reduction (THR) products, or safer cigarettes (SCs), are designed to focus on the reduction of carcinogenic component concentrations in tobacco. Such products are assumed to be able to reduce or eliminate nicotine delivery, and reduce toxicity or mutagenicity of smoke. All LHC products are marketed based on the assumption that the most significant toxicants in cigarette smoke are those that have been identified and studied toxicologically [5]. The makeup and the composition of cigarette smoke have changed remarkably in the past 50 years, resulting decreasing yields of harmful constituents of smokes, such as tar and nicotine [43]. 

#### 2.2.1. Manufacture Approaches

General and selective reduction approaches when manufacturing LHC products are discussed in the review paper [44], *i.e.*, they were employed to mitigate the health risks of cigarettes. For *general* reduction approaches, the industry curtailed all tobacco ingredients via reconstituted tobacco, more efficient filters, burning less tobacco, and more porous tobacco paper. For *selective* reduction approaches, industry tried to reduce individual harmful ingredients via genetic engineering, *i.e.*, primarily benzo[a]pyrene (BaP), tobacco specific nitrosamines (TSNA), phenols, and ciliastats. Most frequently used are reconstitution of tobacco, design of more efficient filter tips, adjustment of reaction temperature, and introduction of electronic cigarettes (e-cigs). 

Other approaches such as blending additives to tobacco to neutralize cancer-causing compounds (Liggett Group, http://www.pbs.org/wgbh/nova/body/safer-cigarettes-history.html) were abandoned because of marketing problems. 

#### 2.2.2. Typical LHC Products

##### Non-Burning Cigarettes

Both Premier^®^ and Eclipse^®^ can be categorized as no-burning cigarettes. In 1988, R. J. Reynolds introduced a high-tech cigarette called Premier^®^. The product is described as a smokeless cigarette that dramatically reduced the cancer-causing compounds inhaled by smokers. The tobacco pellet (not tobacco) was heated instead of burned, thereby producing less smoke and ash than conventional cigarettes. In 1994, R. J. Reynolds began testing the Eclipse^®^ “smokeless” cigarettes, which claimed to reduce secondhand smoke by 85%. The structure and mechanisms of Eclipse^®^ can be found in several papers [45]. Eclipse^®^ is more like an ordinary cigarette than its predecessor Premier^®^ because it contains tobacco or reconstituted tobacco. As the cigarette is heated by lighting the charcoal used as an air heater, heated glycerin aerosols are released to avoid burning of the tobacco. As a result, tobacco emit flavor without generating ash and smoke. Eclipse^®^ emits lower tar levels of cancer-causing compounds than many existing cigarettes. However, it still produces carbon monoxide and nicotine. Additionally, the effect of heating glycerin is carcinogenic (http://www.pbs.org/wgbh/nova/body/safer-cigarettes-history.html). Although unburned tobacco produces less carcinogens than cigarette remains after combustion, several strong carcinogens, e.g., BaP and NNK, were still detected. Specifically, “less carcinogens” indicates less mass and a lower number of carcinogenic chemical species compared to conventional cigarettes.

##### Electrical Heated Cigarettes (EHCs)

Borrowing the same idea that heating rather than combusting tobacco can provide a substantial reduction in many carcinogenic smoke constituents, Patskan and Reininghaus [3] proposed a new design of EHC whose previous product was Accord^®^ (Philip Morris, 1997). This electrically heated cigarette consists of a tobacco mat that surrounds a column of conventional cigarette tobacco filler. This cigarette should be smoked only with a specially designed lighter [3]. They claimed that no sidestream smoke is produced when consuming this product, and there are less carcinogenic matters inhaled per EHC than per conventional cigarette [46]. The product avoids the problem of heating glycerin as required for the Eclipse^®^. The most updated patent of the EHC of Philip Morris, labeled NGP, was designed by Griffin (http://www.freepatentsonline.com/7293565.html). The NGP (2007) is equipped with a more portable heater compared to the old design. Another design of EHC was patented by Felter *et al*. [47] whose design contains a puff sensor.

##### E-Cigarettes

Electronic cigarettes initially emerged in 2003 in China, produced by Beijing SBT Ruyan Technologies and Development with an international patent in 2007 which is reviewed in references [48,49]. An electronic cigarette, also known as e-cigarette, personal vaporizer, or electronic nicotine delivery system (ENDS) [50,51,52,53], is a battery-powered device that provides inhaled doses of nicotine by way of a vaporized solution which then condenses to aerosols. It is an alternative to burned tobacco products, such as cigarettes, cigars, or pipes. For new e-cigarettes, the “smoke” is generated mainly by an atomizer without combustion so that only evaporation and condensation may occur [53]. Nicotine solutions sold separately for use in refillable cartridges are sometimes referred to as “e-liquid” or “e-juice” [48], and commonly contain some amount of flavoring material, with several different flavors available. They consist of nicotine dissolved in propylene glycol (PG) and/or glycerin [49]. In addition to purported nicotine delivery, e-cigarettes also provide a flavor and physical sensation similar to that of inhaled tobacco smoke, while no tobacco, smoke, or combustion is actually involved in their operation. 

Most scientists consider carbon monoxide (CO) and tar to be the main toxic constituents of a cigarette. For example, CO binds to hemoglobin, inhibits respiration and induces atherosclerosis. Based on such a view, e-cigarettes are advertised as “free of primary and second-hand smoke risk” because no CO or tar will be released during the smoking process. However, one must not neglect the health impact of nicotine and other additives. Recently, Williams *et al*. [52] found one brand of e-cigarettes generates aerosols containing micron particles comprised of tin, silver, iron, nickel, aluminum and silicate, as well as nanoparticles containing tin, chromium and nickel, which are elements that cause respiratory distress and disease. Those metals come from the wires inside the cartridge, while silicate particles may originate from the fiber glass wicks. Some observers worry that teenagers may start using e-cigarettes and then advance to smoking actual cigarettes. E-cigarette sales are projected to go into the billions. An up-to-date review of the potential risks of e-cigarettes is discussed by Series [53]. 

#### 2.2.3. Potential Health Risks of LHC Products

Following “less ought to be better”, many papers claim that those LHC products can reduce the health risk of smoking, when compared to conventional cigarettes. For example, Patskan and Reininghaus [3] claimed that their EHC delivered 50% lower amounts of about two-thirds of the 69 smoke carcinogenic constituents than conventional cigarettes with no side-stream smoke. Meckley *et al*. [45] claimed that Eclipse^®^ produces smoke condensates less tumor promoting than conventional cigarette, *i.e.*, Kentucky 1R4F. Most recently, Hatsukami *et al*. [54] announced that reduced nicotine content cigarettes of at least 0.05 mg nicotine yield can lead to reductions in toxicant exposure and can be used as a cessation tool. 

There is no question that the LHC products yield less mass and species in toxic matters [55]. However, since “less mass and species” being yielded is not equal to “less health harm”, *i.e.*, a series of basic problems need to be examined [42]. For example, because of the reduced nicotine content in LHC products compensatory smoking and higher cigarette consumption per day of LHCs may cause more harm [56,57]. Specifically, LHC-smokers developed lung cancer further down into their lungs than conventional cigarette smokers [58]. Laugesen and Fowles [59] evaluated the Marlboro UltraSmooth^®^ which is touted as a cigarette with a new filter to reduce the inhaled carcinogens under more smoker-realistic intensive machine testing. Their study increased the index they used specifically under HCI smoking when normalized per mg nicotine. They found that Marlboro UltraSmooth^®^ increased all toxicants combined for carcinogens and for cardiovascular toxicants, compared to conventional cigarettes. Chen *et al*. [60] executed toxicological analysis and claimed that low-nicotine and nicotine-free Quest^®^ cigarettes do not have less adverse toxicological effects in the laboratory than conventional cigarettes. Side-smoke from Quest^®^ is even more harmful than conventional cigarettes [61,62]. Gan *et al*. [63] claimed that Chinese “Herbal” Cigarettes are as carcinogenic and addictive as regular cigarettes. Concerning e-cigarettes, McCauley *et al*. [64] reported that a 42-year-old woman was admitted with dyspnea induced by continuous consuming e-cigarettes. Other illnesses reported related to e-cigarette consumption includes pneumonia, congestive heart failure, disorientation, seizure, hypotension, possible aspiration pneumonia, chest pain, and rapid heartbeat [65].

In summary, the potential health risks when using LHC products could be as follows:

(a) Compensatory smoking (*i.e.*, stronger puffing) leading to cancer in the deeper lung regions [11,12,13].
(b) Unknown reactions between some components in newly designed filters (or other new additives) may lead to the production of carcinogens or other toxicants.

As LHC products are newly emerging, data and evidence on usage patterns and health impact are still sparse [66]. Thus, scientists and engineers must provide non-biased investigative results and health impact analyses for carcinogens and toxicants discovered in LHC products (see Table 1). 

**Table 1:** Cigarette toxicants and carcinogens and their biomarkers [31,67,68,69]. The superscript * indicates that toxicants exist both in conventional cigarettes and PREPs [46].

Toxicants	Induced Cancer Type	Related Biomarkers
Acetaldehyde *	Lung, nasal	Leukocyte DNA adducts
Acrolein *	Lung	3-HPMA in urine
Benzene *	Lung, leukemia	SPMA in urine
Benzo[a]pyrene *	Lung	1-hydroxypyrene in urine
1,3-Butadiene *	Lung, leukemia, liver	MHBMA in urine
Carbon monoxide *	N/A	Exhaled CO
NNK, NNN *	Lung, nasal, oral cavity, liver, oesophageal, pancreatic, cervical	NNAL and NNN in urine
PAH *	Lung, laryngeal, oral cavity, cervical	1-HOP
Formaldehyde	Lung, nasal	Leukocyte DNA adducts
Nicotine *	N/A	Nicotine, cotinine, 3′-hydroxicotinine and other their glucuronides in urine
Nickel *	Lung, nasal	N/A

### 2.3. Children as the Vulnerable Population Group

Children are uniquely susceptible to toxic aerosols, including cigarette smoke especially due to SHS or ETS [34]. This vulnerability is because of three major reasons: (a) children have disproportionately heavier exposures in relation to body weight than adults for the same amount of toxic aerosols [70]; (b) children are extremely sensitive to these exposures, lacking the ability to metabolize, detoxify and excrete those toxic compounds [71]; and (c) especially small children often reside very close to their parents who may be smokers. The statistics show that in the United States, more than 20% children live with smokers, where especially children living in public housing units endure much higher SHS-exposure than the national average.

Apostolou *et al*. [35] reported that SHS-inhalation increases the blood lead levels in US children, while Knudsen and Kleinstreuer [72] and Proietti *et al*. [73] pointed out that exposure to SHS (and other air-pollutants) may cause disruptive embryonic vascular development and low birth weight. Furthermore, brief acute transient SHS-exposures can also cause adverse effects on respiratory and cardiovascular systems, such as promoting oxidative stress and endothelium dysfunction [37,74,75,76]. Additionally, it is confirmed that SHS-exposures cause a statistically significant increase in other diseases among children, e.g., middle ear disease [34]. 

## 3. Studies of Toxic Aerosols from Inhaled Cigarette Smoke

Apparently, different deposition dosage of toxins in the human lung and blood may pose different chronic and acute effects on pulmonary and cardiovascular organs. At the same time, the deposition of toxins is related to the various patterns of exposure (*i.e.*, duration and intensity over time), breathing waveforms, tobacco smoke characteristics (e.g., droplet size, shape, density, temperature, surface properties, physical and chemical interactions, as well as vapor content), lung morphologies (children *vs*. non-smoking and smoking adults, and the elderly with or without COPD), as well as properties of the mucus-tissue-blood-organ system. Therefore, quantitative and qualitative impact analyses are both valuable ways to assess exposure-dose and potential health-effects by providing toxicologists with reliable, quantitative data sets, as well as qualitative projections. Quantitative approaches include experiments, clinical tests and investigations, semi-empirical models, and computational fluid-particle dynamics (CF-PD) simulations and analyses. Validated modeling results are useful to manufacturers, toxicologists, health-care providers, epidemiologists, and federal regulators alike. 

### 3.1. Experimental and Clinical Investigations

#### 3.1.1. Experimental Studies

Experimental investigations, mostly cigarette smoke exposure studies, are focusing on the impact analyses of tobacco smoke aerosols *in vitro*. Two commonly used experimental setups of cigarette smoke exposure are:

(1) Cigarette Smoke Extract Exposure (CSEE) systems, which collect the CSPs using filters, traps, *etc*. [77].
(2) Whole Smoke Exposure (WSE) systems, in which cell cultures are exposed to smoke directly in an exposure chamber [27,78,79].

For the real-time, puff-by-puff analyses, WSE systems are frequently employed. The use of real-time monitoring techniques can provide precise magnitude of transient concentrations and exposures, as well as calculating mean concentrations and exposures over the time series [33]. Generally, these systems contain:

- A smoking machine to generate and dilute mainstream smoke samples to the experimental chamber, e.g., Walton Smoking Machine (WSM), Borgwaldt RM20S^®^ smoking machine, TE-10z smoking machine, *etc*. [40,78,80,81,82]. The simulated puffing conditions were based on the International Organization for Standardization (ISO).
- An exposure chamber where the smoke and cell cultures (*i.e.*, tissues or physiological fluids samples) interacts with each other [27,78]. Additionally, for clinical study, a human exposure chamber may be applied [40]. It is necessary to carefully regulate the conditions in the chamber, in order to mimic the environment *in vivo*.
- A collection-dilution chamber and a particle size analyzer for aerosol size time-evolution measurements, e.g., Proton-Transfer-Reaction Mass Spectrometry (PTR-MS) [81], Scanning Mobility Particle Sizer (SMPS) [83,84], *etc*.

As most of the smoke constituents are tractable [26], experimental investigations are able to provide comparative data set for computer model validations, and to provide real-time measurements of tobacco smoke aerosol properties, *i.e.*, aerosol droplet size, particle number concentration, and composition [2,11,12,26,84,85,86]. For example, Ingebrethsen *et al*. [2] measured the time-evolution of the diameter of CSPs for different puff regiments. Such findings are useful for future CF-PD model validations, considering hygroscopic growth and Brownian coagulation. Sahu *et al*. [84] investigated the particle size distribution of tobacco smoke for mainstream and (exhaled) sidestream smoke, and the impact of smoking behavior on the particle size distribution of the mainstream smoke.

Furthermore, experimental investigations can assess new toxicants, or compounded health risk effects, generated from new cigarette products [52,78,81,86,87,88,89]. For example, Gordon *et al*. [81] experimentally investigated the effects of cigarette menthol content on the exposure to selected cigarette constituents. Roemer *et al*. [88] assessed the potential effect of using sugars as ingredients in American-blend cigarettes. They claimed that the use of sugars in cigarette tobacco does not increase risk or harm to smokers. Goniewicz *et al*. [89] measured the nicotine level delivered by using an automatic smoking machine, modified to simulate puffing conditions of real e-cigarette users. They found that 50% to 60% of nicotine from a cartridge was vaporized during the first 150–180 puffs, where 0.5 to 15.4 mg of nicotine in vapor form was generated by 20 series of 15 puffs. Using a modified smoking machine, William *et al*. [52] measured selected carcinogens and toxicants in vapors generated from 12 brands of e-cigarettes and discovered that the levels of the toxicants were 9 to 450 times lower than in conventional cigarette smoke. 

Experiments can also provide data of realistic puffing inhalation characteristics related to different tobacco products based on real-time measurements [11,26,85,90,91,92]. For example, Trtchounian *et al*. [11] investigated the puffing strength and aerosol concentration over time when smoking conventional cigarettes or e-cigarettes. Alfi *et al*. [92] analyzed the puff durations and exhalation durations during the use of conventional cigarettes and e-cigarettes. They announced that puff duration for conventional cigarette smokers is 2.4 s in average, while the puff duration is 4.3 s in average for e-cigarette smokers. Longer puff duration may help e-cigarette smokers compensate for a lower delivery of nicotine.

Additionally, experiments can help to establish semi-empirical models to quickly estimate aerosol deposition in the human respiratory system based on the statistics of averaged deposition data obtained *in vivo* and *in vitro* [93]. However, a significant technical challenge that still remains is the development of a standard, machine-based test method for cigarettes that accurately reflects smoke-yield under actual conditions of consumer usage.

#### 3.1.2. Clinical Investigations

Clinical *in vivo* investigations are based on observations of patients or volunteers, where conclusions are drawn according to statistical analyses of data sets and patients’ records [74]. Clinical investigations are important because tobacco product characterizations should not be limited to comparisons under standardized conditions employing smoking machines (as done in laboratory studies) but should also include anticipated conditions of actual use [94]. As experimental investigations are typically designed at the product level, clinical investigations should be at the individual smoker level and/or population level.

Clinical investigations can evaluate the pathology of different toxicants and carcinogens *in vivo* [27,83,85,86,94]. Doses are monitored by the analysis of toxic substances or their metabolites in biological fluids (*i.e.*, biomarkers). Such a method is for evaluating human exposure to toxins [27] to determine the retention of toxins in the human body [83,85,95]. Clinically, Morawska *et al*. [83] reported the total deposition efficiency (TDE) of 36% with a standard deviation (SD) of 10% for second-hand smoke with a CMD of 183 nm, affecting non-smoking individuals. Mcgrath *et al*. [85] and Dickens *et al*. [95] used biomarkers (solanesol) to calculate the TDE of cigarette smoke in seven volunteer smokers. Van Dijk *et al*. [86] revealed the existence of nanoparticles (d_p _= 6 nm to 50 nm) in fresh and undiluted tobacco smoke for moderate smoking regiments. The number of nanoparticles ranged from 2.6e+6 per to 8.8e+9 per cigarette. Van Dijk *et al*. [86] discovered that the particle counts decreased for smaller nanoparticles. This may be because the small nanoparticles have a high potential to quickly agglomerate into larger smoke constituents or to disperse into smaller vapor molecules. Schripp *et al*. [66] measured the time-evolution of e-cigarette aerosol-size distribution during consumption under different chamber conditions.

Clinical investigations can also provide *in vivo* airflow measurements during smoking. However, the measurements are costly and complex to perform, and accurate results are difficult to obtain due to the spatial resolution and tissue attenuation limit. Therefore, replicas of human respiratory systems have been used widely for experiments. Additionally, compared to experimental and CF-PD investigations, it is difficult to obtain *in vivo* local deposition efficiencies and detailed aerosol property variations with time. 

### 3.2. Computational Fluid-Particle Dynamics (CF-PD) Simulation Models

Computational Fluid-Particle Dynamics (CF-PD) simulation models solve governing equations for airflow and particulate phases, providing information on particle and vapor deposition patterns within selected structural elements of the human respiratory systems [96]. Accurate simulations and predictions of airflow structures and related aerosol-phase depositions in realistic models of the human respiratory system are of fundamental importance. Detailed discussions of CF-PD simulation models can be found in several up-to-date review articles [96,97,98,99,100]. 

**Figure 1:**
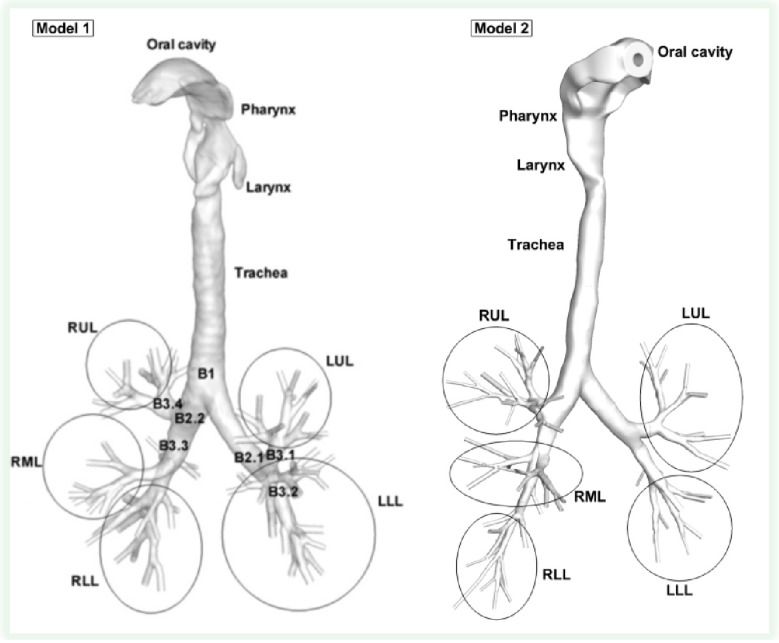
Configuration of subject-specific human airway models (from oral cavity to G9).

Generally, CF-PD simulation models contain three elements:

(1) *Multiphase flow models with relevant physical and bio-chemical processes*. Considering the computational costs and accuracies, the multiphase flow models widely used for simulating tobacco smoke aerosol transport and deposition in human respiratory systems are mostly within the Euler-Lagrange and Euler-Euler frameworks [12,13,97,101]. Specifically, different approaches employed for particulate phases or vapors are as follows:
  (a) Lagrangian approaches employing the point force-and-moment method for transport and deposition simulations of tobacco smoke particulate phases, *i.e.*, particles and droplets [12]. These approaches provide direct descriptions of the particulate flow by tracking the motion of individual particulate entities. The transient airflow field can be solved independently in the Eulerian frame in case of dilute aerosol suspensions.
  (b) Eulerian approaches with enhanced mass transfer for vapors and nanoparticles (less than 50 nm in diameter) in tobacco smoke [13]. Solution of the enhanced mass transfer equation, *i.e.*, Euler-Euler approach, considering inhaled material convection, diffusion, coagulation/aggregation, wall-flux deposition, *etc*.
(2) *Initial and boundary conditions*. Initial and boundary conditions include realistic airflow waveforms as part of smoking behavior, initial particle distribution at the mouth inlet, physical/chemical characteristics of inhaled particles, droplets and vapors, rigid or moving lung airway-wall boundary conditions, lung airway-outlet boundary conditions, *etc*.
(3) *Realistic human respiratory system geometries.* Accurate and realistic human respiratory system models (see examples in Figure 1) compose the necessary precursor for experimental or computational airflow and particle transport/deposition analyses [100]. The human respiratory system ventilation path contains mouth, nose, pharynx, glottis, larynx, trachea, bronchi, bronchioles (including terminal bronchioles (Generation 16) and respiratory bronchioles (Generation 17–19)), and alveoli. Development of a subject-specific model for ventilation of a breathing lung can only be accomplished through multidisciplinary efforts that require expertise in medical imaging, airway geometric reconstruction, computational techniques, pulmonary physiology and medicine, and fluid mechanics [102].

**Figure 2:**
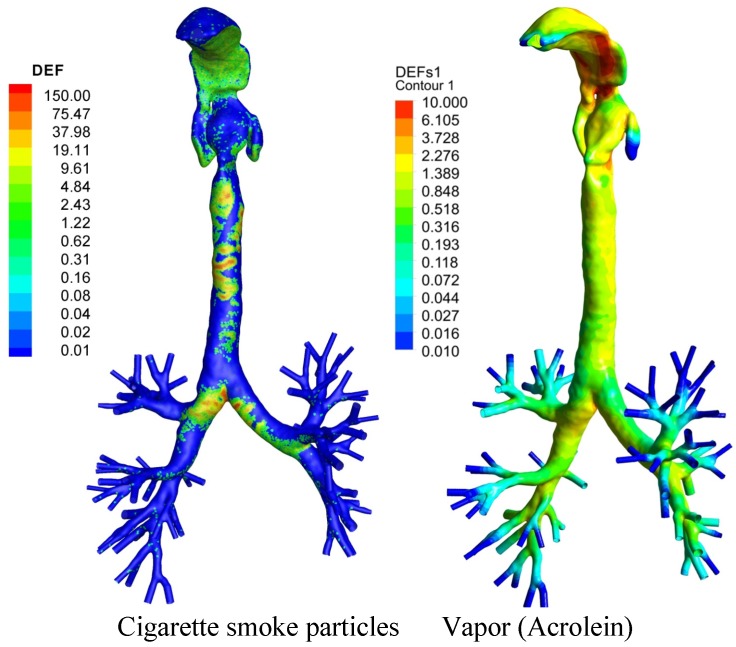
Local deposition patterns of CSPs and acrolein vapor under steady inhalation (Reprinted from [12,13], with permission from Elsevier).

Although the fluid dynamics and biomechanics phenomena occurring in a complete set of realistic pulmonary airways cannot yet be simulated due to the limitations in respiratory-tract imaging and computational resources, CF-PD simulation models are the most promising investigative tools for accurate and realistic impact analyses of toxic tobacco smoke aerosols. Ideally, CF-PD simulation models could combine all physical mechanisms, e.g., condensation and evaporation [12,13,103], Brownian motion and coagulation [97], turbulence dispersion [12,13,104,105], charge effect, *etc*., and reflect the realistic transport and deposition of toxins in subject-specific models of the respiratory system (see sample results in Figure 2). Such detailed results cannot be obtained from experimental or clinical investigations in a cost-effective way. The development of CF-PD simulation models will continue to provide new information for reducing uncertainties in experimental and clinical investigations [106].

### 3.3. Semi-Empirical Models

Semi-empirical models statistically utilize the published experimental and clinical data, combined with theoretical models to provide regional or total deposition efficiencies of tobacco smoke in human respiratory systems. Examples include the Lung Dose Evaluation Program (LUDEP) [82,107], and the Multiple Path Particle Dosimetry (MPPD) model [99]. Rostami [99] reviewed existing semi-empirical models before 2009 with detailed discussions. Kane *et al*. [108] further modified the MPPD model [99,109] by combining the effect of particle diameter and concentration changes due to particle evaporation, hygroscopic growth, and coagulation. Sahu *et al*. [84] employed the particle size distribution obtained by experiments and used the MPPD model to predict the regional and total deposition efficiencies in a human respiratory tract. These models are more computationally economic compared to transient three-dimensional CF-PD models. However, semi-empirical models are not able to provide as many details as CF-PD simulation models. Therefore, with increased computational hardware and software resources becoming available, CF-PD simulation models provide the most promising approaches to obtain detailed knowledge of the deposited amount and distribution of toxins and carcinogens in human airways for pathological assessment and evaluation.

### 3.4. Parameters and Mechanisms Influencing the Deposition of Toxicants and Carcinogens

As mentioned, the transport and deposition of toxicants and carcinogens of tobacco smoke in the human respiratory systems is a complex process which is influenced by many physical mechanisms. They include coagulation, condensation and evaporation, hygroscopic growth, change in composition, and individual puffing behavior [85]. For conventional cigarette smoke, experimental deposition data sets are consistent with classical predictions [110]. It is known that deposition efficiencies (DE) of tobacco smoke aerosols in the lung (60–80%) are greater than expected when smoke particles are in the 150–250 nm range in terms of count median diameter (CMD) [26]. Until now the mechanisms leading to higher DEs have not been fully addressed. For example, it has been experimentally observed that inhaled cigarette smoke particles may grow from 160.1 ± 6.4 nm in diameter to 238.9 ± 16.5 nm by the time they are exhaled, implying a growth factor of 1.5 [85]. The aerosol size development inside the human respiratory system may play an important role to explain the higher DEs. In general, to improve the understanding of the relationships between toxicants and diseases, the following has to be addressed: Definition of potential health risk criteria coupled with efficient potential harm reductions, as well as physical/chemical mechanisms and parameters that influence the transport and deposition of tobacco smoke aerosols. 

#### 3.4.1. Variability in Smoking Behavior

Inhalation of tobacco smoke aerosols is a multi-step process which consists of puffing, mouth hold, inhalation, and exhalation [2,85,90,95,111]. Typically, a smoker first draws a puff-of-smoke into the mouth cavity and holds it with the soft palate closed [95]. However, some smokers may inhale the smoke directly into the lung [111]. It is well observed that the puffing behavior (*i.e.*, volume, duration, inter-puff interval, frequency, and waveform profiles) vary substantially among individuals as well as different tobacco products. 

Different puffing behavior, especially the puffing flow rate will give different transit times of smoke through the shortening length of a cigarette; hence, influencing aerosol size and location distribution, as well as the aerosol composition at the mouth inlet [12,13,26,82,92,95,108]. Influences between puffing behavior and aerosol properties will have a strong impact on the local and total deposition efficiencies in the human respiratory systems. 

Typical puffing characteristics are [108,111]:

- 31 to 86 mL in puff volume.
- 0.9 to 3.0 s in puff duration.
- 18 to 64 s for inter-puff interval.
- 2,100 to 3,800 mL/min in puff flow rate.
- 8 to 16 puffs per cigarette.

**Figure 3:**
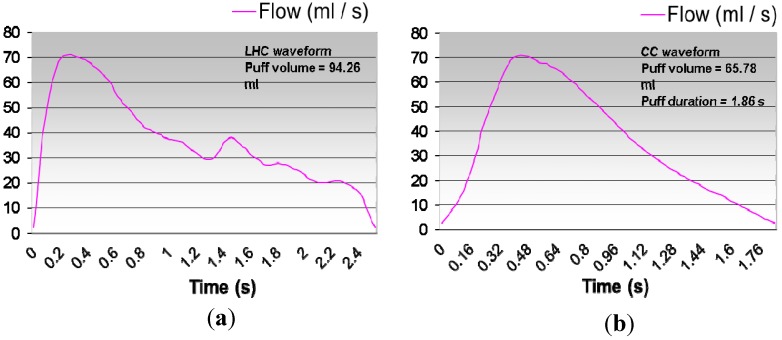
Single puff profiles: (**a**) A typical LHC-puffing waveform (adapted from [13], with permission from Elsevier); (**b**) A typical conventional cigarette puffing waveform.

**Figure 4:**
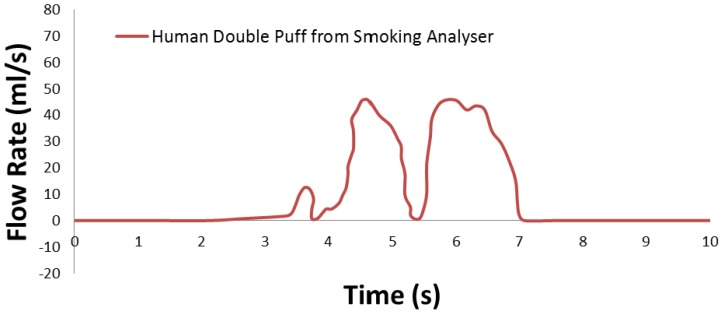
Human double-puff profile (adapted from [85]).

Twenty-five different puffing regiments generated by idealized smoking machines (see Table 2) to reflect “human puffing behavior” are discussed in Ref. [90]. Although the puffing regiments in Table 2 can be utilized in CF-PD simulations for quick analyses, they may not reflect actual puffing behavior. For example, single puffing waveforms for LHC products and conventional cigarettes are shown in Figure 3(a,b). Generally, LHC products (e.g., e-cigarettes) require stronger puffing than conventional cigarettes (see the discussions in the previous sections). Furthermore, double-puffing waveforms were detected by McGrath *et al*. [85], using a pre-calibrated portable smoking analyzer with seven volunteer smokers. A double-puff profile is shown in Figure 4. 

**Table 2:** Smoking machine puffing regimens for conventional cigarettes (Reprinted from [90], with permission from Elsevier).

Cigarette	Smoking Condition	Puff Volume (mL)	Puff Duration (s)	Puff Frequency (min^−1^)
EHCSS-K6	LOW4	40	1.2	4.0
	MED1	78	1.8	1.0
	MED2L	78	1.0	2.0
	MED2U	78	2.6	2.0
	MED4	78	1.8	4.0
	HIGH1	126	2.6	1.0
	HIGH 2L	126	1.8	2.0
	HIGH2U	126	3.4	2.0
	EXTREME	126	3.8	2.0
EHCSS-K3	LOW	40	1.2	2.0
	MED	78	1.8	2.0
	HIGH	126	2.6	2.0
	EXTREME	126	3.7	2.0
M6UK	LOW	26	0.8	1.9
	MED	50	1.4	1.7
	HIGH	66	1.7	2.0
M6J	LOW	15	0.7	0.9
	MED	39	1.1	1.0
	HIGH	55	1.5	1.2
PM1	LOW	29	0.9	3.6
	MED	50	1.4	3.4
	HIGH	64	1.6	4.0
Lark1	LOW	29	0.9	3.0
	MED	42	1.2	2.5
	HIGH	62	1.6	2.6

For post-puff ventilation behavior, St Charles *et al*. [91] tested 74 established smokers, and reported a mean inhalation volume ranging from 649 to 841 mL, mean lung exposure times from 4.5 to 5.6 s, and a mean inhalation tidal ratio of 1.77. 

#### 3.4.2. Mechanisms Influencing Time-Evolution of Aerosol Size

Mechanisms of inhaled toxic aerosols which may influence aerosol size over time include multi-component droplet/vapor compositions and interactions, condensation and vaporization of droplets/vapors, coagulation effects among particles, droplet-cloud formation and motion, particle charge effect, *etc*. Several of those mechanisms strongly influence the local and total deposition efficiency of toxicants and carcinogens. 

##### Evaporation or Hygroscopic Growth Effect

The evaporation hygroscopic growth of a droplet is driven by the chemical potential difference of constituent species between the droplet and the surrounding gas phase. The hygroscopic growth of cigarette smoke droplets in the human respiratory system can be significant, and hence may notably alter the behavior and fate of the dispersed phase [105]. For conventional cigarette smoke particles (CSPs), a number of *in vivo, in vitro* and numerical studies have been performed. For example, Hicks *et al*. [112] reported that the inhaled dense mainstream CSPs may experience a diameter-increase up to 1.7 times at exhalation. Li and Hopke [113] measured an average growth rate of 1.54 for mainstream CSPs with initial diameters ranging from 150 nm to 400 nm at a relative humidity (RH) of 99.5%. Robinson and Yu [110,114] and Longest and Xi [105] reported an equilibrium growth ratio of 1.4 to 1.7 for mainstream CSPs when the RH approaches 100%. In summary, the hygroscopic growth of conventional CSP is around 1.5 at body temperature and humidity conditions (*i.e.*, T = 37 °C and RH = 99.5%) [115]. 

The possible size-changes of LHC products may differ from the CSPs [12]. Therefore, the understanding and accurate description of species heat and mass transport processes are key for the simulation of droplet vaporization or growth. There are six groups of CF-PD models based on various assumptions describing the heat and mass transfer associated with real-world evaporation scenarios [116]. Specifically, the CF-PD modeling assumptions for the six groups of mathematical models are as follows.

- Group 1: Assuming that the droplet surface temperature is uniform and does not change with time.
- Group 2: Assuming that no temperature gradient and species mass fraction gradient exist inside the droplets, *i.e.*, infinite thermal conductivity and mass diffusivity in the liquid phase.
- Group 3: Taking into account the temperature gradient and mass fraction gradient inside droplets without considering the recirculation inside droplets (Hill’s vortex) which would enhance the effective thermal conductivity of the liquid.
- Group 4: Based on the Group 3 conditions, taking into account the recirculation effect by introducing a correction factor to the liquid thermal conductivity.
- Group 5: Describing the recirculation effect by simulation of the internal vortex dynamics.
- Group 6: Full solution of the multi-phase Navier-Stokes equations.

Models of Group 1 are suitable for analytical analysis due to their highly simplified assumptions. Group 5 and Group 6 have not been widely employed because of their complexities and high computational costs. Models of Groups 2 to 4 are widely used in most practical applications. 

Recently, Zhang *et al*. [12] investigated numerically the size evolution and deposition of conventional cigarette and LHC product droplets in a subject-specific airway model. They validated their numerical model with previous *in-vivo* and *in-vitro* studies and found that the hygroscopic growth has no significant impact on droplet size and deposition. Furthermore, Kim *et al*. [117] validated the hygroscopic CF-PD model with experimental data for droplet growth on the submicron scale. They evaluated the hygroscopic effect in the nasal airway of a 5-year-old child for initially mono-disperse 200 nm droplets. Additionally, Sazhin *et al*. [118] developed a kinetic model for droplet heating and evaporation into a high-pressure environment, which takes into account the effects of inelastic collisions, a non-unity evaporation coefficient, and temperature gradients inside the droplets. However, their kinetic model is computationally extremely expensive and hence is presently not suitable for large-scale lung aerosol dynamics simulations. 

In light of the complex multi-component aerosols in conventional cigarettes and LHC products, the mass transfer for each component may contribute to the total mass change of the droplet. Therefore, considering the compromise between accuracy and computational cost, the vaporization/hygroscopic growth of tobacco smoke aerosols can be given as [12]:

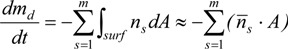
(1)
where *m_d_* is the droplet mass and *n_s_* is the mass flux of each chemical species at the shrinking/growing droplet surface, which can be calculated by:

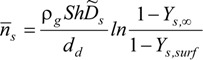
(2)


Here, *Y_s, surf_* and *Y_s, __∞_* are the gas-phase mass fractions of each component on the droplet surface and far from the droplet, respectively. The surface mass fraction (concentration) of species on the droplet surface can be calculated with a modified Raoult’s law, *i.e.*,:

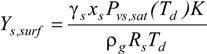
(3)


In the above expression, *γ_s_* is the activity coefficient of species *s*, which is a correction for interactions in the liquid phase between the different species molecules; *x_s_* is the liquid-phase mole fraction of the species of interest, *R_s_* is the species gas constant, *T_d_* is the droplet temperature, and *P_sv,sat_(T_d_)* is the temperature dependent saturation pressure of the species. The mole fraction of each species in the droplet can be calculated with the following equation:

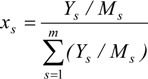
(4)
where *Y_s_* and *M_s_* are mass fraction and molecule weigh of species s.

In addition, in Equation (3), *K* is the correction factor considering the Kelvin effect for small droplets, which is given as:

*K = exp[4σM /(R_u_)ρ_d_d_d_T_d_)]* (5)

where σ is the surface tension at the droplet surface (which is about 0.07 Nm^−1^ for water at 303 K), *M* is the molar mass of the vapor molecules, and *R_u_* is the universal gas constant.

The coupled heat transfer equation for liquid droplets reads:

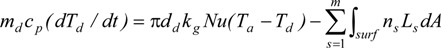
(6)
where *c_p_* is the liquid specific heat; *d_d_* is the droplet diameter; *k_g_* is the thermal conductivity of gas mixture; *T_d_* and *T_a_* are the temperature of droplet and surrounding air, respectively; *L_s_* is the latent heat of vaporization.

The non-continuum effects (Knudsen number correction) can be considered by including correction factors *C_m_* and *C_T_* in the vaporization and heat transfer equations for submicron NGP droplets or conventional CSPs, *i.e.*, Equations (1) and (6) are replaced by [119,120]:

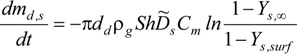
(7)

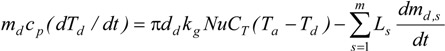
(8)
where:

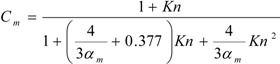
(9)

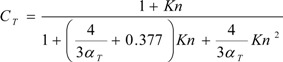
(10)


Here, *Kn* is the Knudsen number, *α_m_* and *α_T_* are the mass and thermal accommodation coefficients, respectively. 

##### Particle-Particle Interactions

Smoke particles in tobacco mainstream are typically 150–250 nm in CMD with a concentration of 10^12^ particles per cigarette smoked. In such a dense air-droplet suspension, particle-particle interactions in terms of Brownian coagulation and cloud formation may occur. Thus, particle-particle interaction must be considered as the primary factor which will cause aerosol size and particle number change, thereby influencing the aerosol transport and deposition in human respiratory systems. For SHS or ETS, as the concentration is not high, interactions between particles can be neglected [121]. 

**Figure 5:**
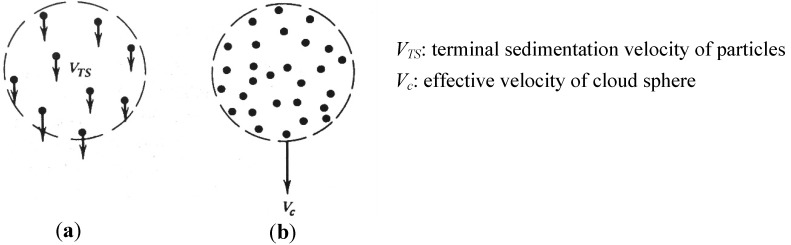
General sketch of: (**a**) Individual particle motion (**b**) Cloud motion (adapted from [127], with permission of John Wiley & Sons, Inc.).

*Particle Cloud Formation Effect.* A “cloud” is defined as a very larger number of particles surrounded by clear fluid [112,113,114,115,116,117,118,119,120,121,122,123,124,125,126]. A cloud of droplets can be treated as a single droplet (*i.e.*, a fluid sphere as shown in Figure 5(a,b)) which has a different density and viscosity than the each individual particle, with no surface tension between this effective droplet and the surrounded fluid [125]. 

The conditions for the onset of cloud formation can be estimated by evaluating the parameter *G*, which is defined as the ratio of the cloud settling velocity to the individual particle settling velocity [128]:

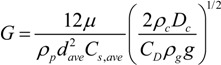
(11)
Here *ρ_p_* is the density of particles, *ρ_g_* is the density of surrounding gas, *ρ_c_* is the cloud density, *i.e.*, the particle mass concentration, *C_s_* is the slip correction factor for the mean particle diameter, *d_ave_*, *C_D_* is the drag coefficient of the cloud, g is gravity and µ is the viscosity of the surrounding gas. If *G* >> 1, the cloud motion effect is essential. 

The cloud formation (or colligative) effect may contribute to the enhanced deposition in the upper airway region for high-concentrated smoke aerosol particles, *i.e.*, the surrounding gas travels effectively around instead of through the droplet-cloud [12,114]. Effectively, the fall velocity of the cloud is greater than that of individual droplets, which enhances particle-deposition due to inertial impaction.

**Figure 6:**
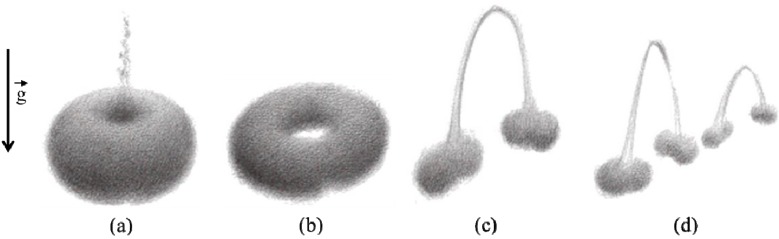
Four phases of particle cloud evolution during settling (adapted from [125]): (**a**) the initial acceleration phase; (**b**) the torus shape phase; (**c**) the break-up phase 1; (**d**) the break-up phase 2.

However, it should be noted that a complete description of cloud behavior in different flow fields is complicated. As shown in Figure 6(a)–(d), the general cloud evolution during, say, sedimentation can be separated into four phases [122,123,124,125,126,127,128,129]:

(1) The initial acceleration phase: the cloud accelerate to its maximum velocity, during which the particles circulate in a toroidal vortex (Hill’s vortex) inside the cloud, in a manner similar to the heavy fluid inside a droplet descending in a lighter fluid. Chaotic fluctuations due to the particle-particle and particle-flow interactions will cause some particles to start cross the boundary of the closed surfaces (see Figure 6(a)).
(2) The torus shape phase: The initial spherical shape cloud gradually evolves into a flattened oblate shape and eventually a torus due to the “leaking” of particles and the toroidal circulation motion (see Figure 6(b)).The torus expands until it reaches a critical aspect ratio.
(3) The break-up phase: After the torus expands to the critical aspect ratio, it becomes instable and the cloud start to break into two and further four smaller particle clouds (see Figure 6(c) and (d)).

According to the complexity of the airflow regimes inside human respiratory systems (see Figure 1), the evolution of tobacco smoke particle cloud during smoking is still unclear and lack of investigations [12]. The cloud behavior of tobacco smoke droplets is currently being approximated by assuming a suitable cloud diameter in the particle trajectory equation for sedimentation and impaction [12,114,129,130]. These models pre-define particle clouds as spheres with prescribed diameters, compare the settling velocity of a cloud with a typical individual particle, and determine the onset of cloud motion when the settling velocity of an individual particle is smaller than that of the cloud. Specifically, Martonen and Musante [130] adopted a “fluid sphere” model by treating the cloud as a fluid sphere moving in a fluid with different viscosity. In this model internal circulation can be considered, which is produced by tangential forces as the cloud moves through the surrounding air. Robinson and Yu [114] presented a model for treating the CSP cloud as a solid sphere. Similar to the fluid sphere model, particles move either as individual particles or as clouds without any intermediate state. Broday and Robinson [129] adopted Brinkman’s effective medium approach and regarded the cloud as a porous medium. In this model the cloud’s permeability changes according to the cloud’s “solid volume” fraction; thus, the transition between individual particles and a cloud is smoother. The limitations of the models mentioned above are obvious. First spherical shape is assumed, which could hardly happen in the complex geometry with turbulent flow and secondary velocity fields; second, a diameter is arbitrarily assigned to the cloud in a certain generation of the lung airways with little reasoning; third, fluid flow through the cloud area is either neglected or highly simplified.

*Brownian Coagulation Effect*. Coagulation effect was claimed as the primary mechanism for changing the mainstream smoke particles’ size [2,26,84,108]. Experimentally, McAughey *et al*. [26], Dickens *et al*. [95], and Kane *et al*. [108] indicated that with increasing filter ventilation and lower puff flow rates, a consequence of increasing aerosol diameters induced by coagulation in the cigarette rod was observed. Asgharian *et al*. [131] claimed a significant coagulation was observed at initial puff concentration of near 10^11^ cm^−3^. At such a concentration, CSPs reach their final size in 10 s. As a benchmark reference, Ingebrethsen *et al*. [2] executed detailed investigations on CSPs coagulation during different smoking breathe stages, *i.e.*, puffing, mouth hold, and inhalation. They found rapid coagulation of CSPs started from earlier in the puff due to the strong drawing airflow into the mouth cavity. After the mouth hold stage, coagulation proceeds at a reduced rate during the inhalation because of the dilution of the smoke concentration. They also found that CSPs less than 10 nm in diameter are greatly reduced in number during the unavoidable mouth coagulation during puffing and virtually eliminated after 1 s of mouth hold. Numerically, introducing Hinds’ [128] and Friedlander’s [132] work on particle coagulation by Brownian diffusion; Kane *et al*. [108] modified the MPPD model with particle coagulation effect.

A thorough understanding of the fundamental properties of the Brownian coagulation ratio of tobacco smoke aerosol with different particulate size distributions plays an important role in the quantitative estimation of particle deposition. It has been found that for ultrafine particles, the coagulation should not be ignored when particle number concentration is more than 2.0e+4 cm^−3^ [133]. Simply, the particle size change due to coagulation can be calculated with empirical (or analytical) coagulation rates [128]:

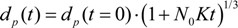
(12)


Here, *t* is the aging time, *N_0_* is the number concentration at *t* = 0, and *K* is the coagulation coefficient. The measured coagulation rate for mainstream CSPs are around 6~12 × 10^−10^ mL/s [134]. A more generalized particle dynamical equation [132,135] can be employed for future coagulation modeling. Another way to numerically modeling Brownian coagulation between aerosol particles is the cohesion model in discrete element method (DEM) [97]. 

##### Charged-Particle Effect

Electrically charged particles are likely to deposit on the surface due to electrostatic precipitation [99]. Experiments showed that cigarette smoke contains charged particles [110]. But Robinson and Yu [114] found with whole-lung modeling that the charge effect is negligible compared to the stable charge-neutral case. It should be noted that charges of CSPs are due to the combustion process. Thus, the process to generate smoke aerosols is essential to determine whether charges will exist or not. The descriptions of both image-charge force and space-charge force can be found in [136]. 

## 4. Conclusions and Future Directions

### 4.1. Summary

The potential health risks of different toxins and carcinogens when inhaling tobacco smoke are reviewed. Advantages and disadvantages of different approaches (*i.e.*, experimental, clinical, and CF-PD) for quantitative and qualitative impact analyses are outlined. The three major factors which influence the transport and deposition of toxins and carcinogens of tobacco smoke in lung-airways include:

- Inter-subject variability in respiratory tract geometry;
- Air-particle inlet conditions; and
- Type and properties of inhaled toxic aerosol.

Of special interest are the impact of realistic puffing waveforms on lung-aerosol penetration and deposition, availability of reliable experimental and clinical data sets for more comprehensive computational analyses, the differences in smoke content and lung-aerosol dynamics of conventional cigarettes and so-called “less-harmful” cigarettes, and the health impact of second-hand smoke inhalation on children. The high variability in types of cigarettes and smoke samples, as well as the scarcity of reliable experimental data sets for comprehensive CF-PD simulations present major challenges for researchers to predict the health impact of smoke-inhalation on different population groups in a realistic and accurate fashion. 

### 4.2. Future Directions for Experimental Studies

(1) *High-resolution puff-by-puff measuring techniques for dense tobacco smoke.* Present techniques and devices (e.g., Scanning Mobility Particle Sizer (SMPS), Optical Particle Counters (OPC), and Aerodynamic Particle Sizer (APS)) for measuring particle diameter time-evolution dynamics and deposition efficiency require *diluted* aerosol suspensions; thus, any influence of coagulation in the original dense suspensions is diminished [87]. Specifically, because most of the measurement techniques are low-time resolution, so that in order to capture aerosol particulate evolution, the high number concentration of the tobacco smoke need to be diluted [86]. Hence, high-time resolved, puff-by-puff measuring techniques have to be developed, and future work should focus on improving real-time quantitative measurements of key toxicants and nicotine inhaled and exhaled. This will allow improved estimates of regional depositions of toxic chemical species and particles to better improve dosimetry and quantitative risk assessment.
(2) *Pathological Biomarkers and Mechanisms*. Although the causal relationship between smoking and several diseases has been well established, there is still little understanding of the underlying mechanisms. Furthermore, the health impact of the release of the volatile organic compounds from the “e-juice” and the release of e-cigarette particulate phase into the indoor environment are still mostly unknown [66]. Although many carcinogen biomarkers have been identified, difficulties exist in tracking them in the human lung airways and beyond. For example, amounts of strong carcinogens [31] are very limited per cigarette (*i.e.*, 1–200 ng per cigarette). Therefore, available biomarkers need to be identified for further investigations.

### 4.3. Future Directions for CF-PD Simulations

(1) *Accurate Image Processing:* Currently, 100% accurate realistic 3-D imaging and modeling of the entire human respiratory system is unrealistic for several reasons: (a) the resolution of CT/MRI is not sufficiently high to capture lung airway geometries on a small scale, *i.e.*, airways exceeding generation 6 (G6); (b) the lung consists of 223 airways plus millions of alveoli; (c) *in vivo* measurements are difficult because the whole respiratory system geometry is time dependent according to the human respiratory movements [137]. Accurate and high-resolution image processing techniques of the future will be the cornerstone of CF-PD simulations of the transport and deposition of toxicants and carcinogens in the whole human respiratory system.
(2) *Realistic smoking inlet conditions.* Most CF-PD simulations assumed that the aerosols are directly inhaled into the lung (e.g., without considering the closing of the soft palate during puffing). Thus, the following three steps of smoking, *i.e.*, mouth hold, inhalation and exhalation must be accurately modeled due to their strong influences on aerosol size evolution [26,85]. Furthermore, the puffing strength is not constant during the consumption of one cigarette [11], *i.e.*, it increases as the puff number increases.
(3) *Time-evolved aerosol size distribution release at the mouth inlet.* Presently, assumed aerosol-size distributions at the mouth inlet, employed as boundary conditions for CF-PD simulations, are not time-developed. As it is evident from experiments, most smoke constituents feature a continuous increase from the first puff to the last puff. Also, for e-cigarettes (e.g., NJOY^®^), the aerosol concentration decreases rapidly as the puff number increases during smoking [11]. Furthermore, the transport of droplets probably suffers coalescence which would break the assumption of monodisperse particles. In this case the microscopic mechanism that lead to droplet coalescence need to be investigated and incorporated in the model, as well as the resulted polydisperse distribution. For accurate numerical prediction of the deposition patterns, time-evolved aerosol-size distributions of CSPs based on accurate experimental measurements are necessary.
(4) *Fluid-Structure Interaction.* The assumption of rigid walls is a potentially misleading approximation considering that moving lung airway walls will influence the airflow characteristics and hence airflow-particle interaction in the near-wall region, thereby altering the deposition patterns of the particles. Thus, fluid-structure interaction (FSI) analysis should be introduced for the solution of airflow quantities (*i.e.*, velocity, pressure and shear stress) impacted by continuously deforming geometries (*i.e.*, near-mesh displacement and velocity), as well as the influence on the local DE of CSPs.
(5) *Coupled Droplet-Vapor Interaction.* Currently, the vaporization of droplets with toxicants and vapor transport are uncoupled [62,63]. That is, except for water, the realistic vapor concentrations are ignored for the vaporization of officially identified toxicants in CSPs, while the vaporization mass is not considered in the mass transfer equation either. However, for more accurate modeling, the effect of coupled vaporization and vapor transfer should be investigated. Thus, the local vaporized mass of the objective toxicant has to be added to its vapor transport equation as a source term, while realistic vapor concentrations have to be employed in simulating the vaporization of species. In addition, the local and segmental mass loss due to wall deposition should be considered.
(6) *Nanoparticles/vapors in Tobacco Smoke.* During the smoking of tobacco, some constituents on the nano-scale penetrate the pulmonary alveoli and enter via lymph and/or blood circulation other organs [138]. Thus, a realistic and accurate multi-compartment model for deposited constituent mass transfer into systemic regions is a valuable and cost-effective tool for toxicologists and others to establish dose-response-effect relationships and generate new physical insight and reliable, quantitative data sets [14,15].
(7) *Particle Shape Effect.* The filters of typical commercial cigarettes contain microscopic, needle-shaped shards of glass wool (like fiberglass insulation) which escape into the mouth and throat, and then lodge with tobacco tar in the lung tissue, surrounding the alveoli and lead to COPD, emphysema and eventually lung cancer. Numerous studies have demonstrated that the fiber aspect ratios as well as fiber durability are critical factors involved in pathogenicity [97]. Therefore, it is important to extend CF-PD modeling and accurately describe the orientation and transport of inhaled glass fibers.

## Abbreviations

1-HOP: 1-Hydroxypyrene
3-HPMA: 3-Hydroxypropylmercapturic Acid
APS: Aerodynamic Particle Sizer
CF-PD: Computational Fluid-Particle Dynamics
COPD: Chronic Obstructive Pulmonary Disease
CMD: Count Median Diameter
CORESTA: Cooperation Centre for Scientific Research Relative to Tobacco
CSEE: Cigarette Smoke Extract Exposure
CSP: Cigarette Smoke Particles
DE: Deposition Efficiency
DEM: Discrete Element Method
e-cigs: Electronic Cigarettes
EHC: Electronic Heating Cigarettes
ENDS: Electronic Nicotine Delivery Systems
ETS: Environmental Tobacco Smoke
FTC: Federal Trade Commission
FSI: Fluid-Structure Interaction
ISO: International Organization for Standardization
LDE: Local Deposition Efficiency
LHC: Less Harmful Cigarettes
LLL: Left Lower Lung
LUL: Left Upper Lung
LUDEP: Lung Dose Evaluation Program
MHBMA: Monohydroxy-3-Butenyl Mercapturic Acids
MPPD: Multiple Path Particle Dosimetry
MS: Mainstream Smoke
NNAL: 4-(Methylnitrosamino)-1-(3-Pyridyl)-1-Butanol
NNK: 4-(Methylnitrosamino)-1-(3-Pyridyl)-1-Butanone
NNN: *N*'-Nitrosonornicotine
OPC: Optical Particle Counters
PAH: Polycyclic Aromatic Hydrocarbons
PG: Propylene Glycol
PREP: Potential Reduced Exposure Product
PTR-MS: Proton-Transfer-Reaction Mass Spectrometry
RH: Relative Humidity
RLL: Right Lower Lung
RML: Right Middle Lung
RUL: Right Upper Lung
SC: Safer Cigarettes
SD: Standard Deviation
SHS: Second-hand Smoke
SMPS: Scanning Mobility Particle Sizer
SPMA: S-Phenylmercapturic Acid
TDE: Total Deposition Efficiency
THR: Tobacco Harm Reduction
TPM: Total Particulate Matter
TSNA: Tobacco Specific Nitrosamines
WSE: Whole Smoke Exposure
WSM: Walton Smoking Machine
